# Mineral Stress Drives Loss of Heterochromatin: An Early Harbinger of Vascular Inflammaging and Calcification

**DOI:** 10.1161/CIRCRESAHA.124.325374

**Published:** 2025-01-22

**Authors:** Chin Yee Ho, Meng-Ying Wu, Jirapath Thammaphet, Sadia Ahmad, James Ho C.S., Lilia Draganova, Grace Anderson, Umesh S. Jonnalagadda, Robert Hayward, Rukshana Shroff, Wilson Tan Lek Wen, Anja Verhulst, Roger SY. Foo, Catherine M. Shanahan

**Affiliations:** 1 British Heart Foundation Centre for Research Excellence, School of Cardiovascular and Metabolic Medicine and Sciences, James Black Centre, King’s College London, United Kingdom (C.Y.H., M.-Y.W., J.T., S.A., L.D., G.A., R.H., C.M.S.).; 2 Nanyang Technological University, Singapore (J.H.C.S., U.S.J.).; 3 Nephrology Unit, Great Ormond Street Hospital and University College London Institute of Child Health, United Kingdom (R.S.).; 4 Cardiovascular Disease Translational Research Programme, National University of Singapore Yong Loo Lin School of Medicine (W.T.L.W., R.F.).; 5 Laboratory of Pathophysiology, Department of Biomedical Sciences, University of Antwerp, Wilrijk, Belgium (A.V.).

**Keywords:** aging, heterochromatin, lamins, muscle, smooth, vascular

## Abstract

**BACKGROUND:**

Vascular calcification is a detrimental aging pathology markedly accelerated in patients with chronic kidney disease. PLA (prelamin A) is a biomarker of vascular smooth muscle cell aging that accelerates calcification however the mechanisms remain undefined.

**METHODS:**

Vascular smooth muscle cells were transduced with PLA using an adenoviral vector and epigenetic modifications were monitored using immunofluorescence and targeted polymerase chain reaction array. Epigenetic findings were verified in vivo using immunohistochemistry in human vessels, in a mouse model of inducible prelamin A expression, and in a rat model of chronic kidney disease–induced calcification. Transcriptomic and chromatin immunoprecipitation followed by sequencing analyses were used to identify gene targets impacted by changes in the epigenetic landscape. Molecular tools and antibody arrays were used to monitor the effects of mineral dysregulation on heterochromatin, inflammation, aging, and calcification.

**RESULTS:**

Here, we report that depletion of the repressive heterochromatin marks, H3K9me3 (histone H3, lysine 9, trimethylation) and H3K27me3 (histone H3, lysine 27,trimethylation), is an early hallmark of vascular aging induced by both nuclear lamina dysfunction and dysregulated mineral metabolism, which act to modulate the expression of key epigenetic writers and erasers. Global analysis of H3K9me3 and H3K27me3 marks and pathway analysis revealed deregulation of insulin signaling and autophagy pathways as well as cross-talking DNA damage and NF-κB (nuclear factor κB) inflammatory pathways consistent with early activation of the senescence-associated secretory phenotype. Expression of PLA in vivo induced loss of heterochromatin and promoted inflammation and osteogenic differentiation which preceded aging indices, such as DNA damage and senescence. Vessels from children on dialysis and rats with chronic kidney disease showed prelamin A accumulation and accelerated loss of heterochromatin before the onset of calcification.

**CONCLUSIONS:**

Dysregulated mineral metabolism drives changes in the epigenetic landscape and nuclear lamina dysfunction that together promote early induction of inflammaging pathways priming the vasculature for downstream pathological change.

**Novelty and Significance**

**What Is Known?**

- Vascular calcification is a detrimental pathology that tracks strongly with chronological age.
- Vascular calcification is markedly accelerated in patients with metabolic disorders such as mineral dysregulation in chronic kidney disease.
- Nuclear lamina dysfunction is a hallmark of vascular aging and calcification.

**What New Information Does This Article Contribute?**

- Nuclear lamina dysfunction and metabolic stress induce rapid loss of heterochromatin reiterating features of the senescent epigenetic landscape.
- Loss of heterochromatin is associated with deregulation of metabolic processes and cross-talking of inflammatory and DNA damage response pathways linked to senescence.
- Loss of heterochromatin primes the vessel wall for accelerated inflammaging and calcification.

Aging and its associated pathologies are overwhelmingly the next challenge in cardiovascular disease. Dysregulated mineral metabolism is emerging as a key driver of vascular aging. We show that loss of heterochromatin is an early event in vascular aging driven by nuclear lamina dysfunction and elevated calcium and phosphate. Loss of heterochromatin deregulates key metabolic and cellular homeostatic pathways that are signatures of cellular and organismal aging and primes the vessel wall for subsequent calcification. Mitigating metabolic risk factors and targeting epigenetic modifications with drug or lifestyle interventions are new avenues for treatment of intractable age-related pathologies.

**Meet the First Author, see p 356**

**Editorial, see p 400**

Aging is the strongest risk factor for cardiovascular disease.^1^ Although long considered unmodifiable, increasing evidence suggests aging is associated with specific pathological changes in tissue homeostasis and the accumulation of senescent cells which act to accelerate organ dysfunction in part, by promoting low-level, chronic, and sterile inflammation termed inflammaging.^2–4^

Vascular calcification is a cell-mediated, ubiquitous aging pathology that closely tracks with chronological age and increased cardiovascular risk.^5,6^ Vascular smooth muscle cells (VSMCs) orchestrate the mineralization process by undergoing phenotypic change towards an osteogenic-like state.^7^ Senescent VSMCs exhibit enhanced osteogenic differentiation driven by DNA damage signaling and activation of the senescence-associated secretory phenotype (SASP) where the coordinated secretion of proinflammatory cytokines and chemokines act in a paracrine manner to further enhance osteogenic differentiation.^8–16^

A link between VSMC senescence and calcification is also evident in the premature aging disorder Hutchinson-Gilford progeria syndrome where vascular calcification is a predominant pathology.^17,18^ Hutchinson-Gilford progeria syndrome is caused by mutations in the genes encoding the nuclear lamina protein lamin A or its processing enzyme FACE1, leading to the accumulation of mutated (progerin) or intact forms of the lamin A precursor protein, PLA (prelamin A).^19,20^ PLA interferes with nuclear organization and function including DNA damage repair, thereby acting to accelerate cellular senescence.^11–13,21^ Importantly, we have previously shown that PLA invariably accumulates as VSMCs approach replicative senescence in vitro,^13^ while lamin A precursors have also been shown to accumulate in the arteries of aged and calcified patients strongly suggesting a causal relationship between nuclear lamina dysfunction and vascular calcification during normal aging.^12,13,18,22^

Emerging epidemiological evidence gleaned from large patient biobank resources have demonstrated that elevated serum phosphate and calcium are the greatest risk factors for the development of vascular calcification and mortality, even when levels of these molecules are within the normal range.^6^ Consistent with this observation, patients with chronic kidney disease (CKD) develop markedly accelerated vascular calcification that is uncoupled from chronological age.^23^ This is most pronounced in children and young adults on dialysis who, in response to dysregulated mineral metabolism, develop extensive vascular calcification leading to cardiovascular mortality equivalent to octagenarians.^24,25^ How metabolic disturbances promote vascular calcification is a matter of fierce debate. However, evidence from both human and animal studies suggest that dysregulated mineral metabolism can promote DNA damage, premature senescence, and consequent inflammaging and calcification.^13,15,26,27^ The detection of PLA in the calcified arteries of children with CKD suggests that nuclear lamina dysfunction may also contribute to metabolically accelerated vascular calcification.^13^

Previous studies have identified strong links between nuclear lamina dysfunction and epigenetic change. Indeed, cells from patients with Hutchinson-Gilford progeria syndrome show pronounced differences in histone modifications, typically with loss of the heterochromatin marks, H3K9me3 (histone H3, lysine 9, trimethylation) and H3K27me3 (histone H3, lysine 9, trimethylation), and redistribution of euchromatin.^28,29^ Epigenetic drift and heterochromatin loss are also key features of normal aging,^30^ but it is unknown what causes these specific epigenetic changes and how and when they contribute to senescence and aging pathologies.^31^ The cyclin dependent kinase inhibitor 2A (*CDKN2A*^INK4A)^ gene locus, which encodes the key senescence marker p16, is a genome-wide association study locus for cardiovascular disease and is subject to epigenetic regulation.^32^ PRC (polycomb repressive complex proteins 1/2) can repress p16 expression in young, proliferating cells by inducing high occupancy of H3K27me3, whereas, conversely, its derepression in senescent cells is associated with loss of H3K27me3.^33^

Here, we explored unifying epigenetic mechanisms involved in nuclear lamina dysfunction and VSMC aging and their links to dysregulated mineral metabolism, which plays a critical role in vascular calcification. We show that loss of heterochromatin is an early and ubiquitous feature of vascular aging preceding the onset of calcification. This epigenetic signature could be recapitulated by accumulation of PLA or by dysregulated mineral homeostasis which acts to perturb the innate balance between epigenetic writers and erasers, resulting in a decrease in repressive histone modifications and leading to activation of p16 and multiple SASP genes, mirroring features of senescent cells. Our results link metabolic stress and nuclear lamina–induced global epigenetic change to the establishment of a proinflammatory tissue microenvironment that is prone to mineralization.

## Methods

### Data Availability

All supporting data are available within the article and its Supplemental Material. Please refer to the Supplemental Methods for detailed experimental methods. Research materials listed in the Methods are included in the Major Resources Table in the Supplemental Material. All data and materials have been made publicly available at the (National Center for Biotechnology Information [NCBI] Sequence Read Archive serial number GSE234290) and can be accessed at https://www.ncbi.nlm.nih.gov/geo.

## Results

### Nuclear Lamina Abnormalities Change the Repressive Chromatin Environment

**Figure 1.**
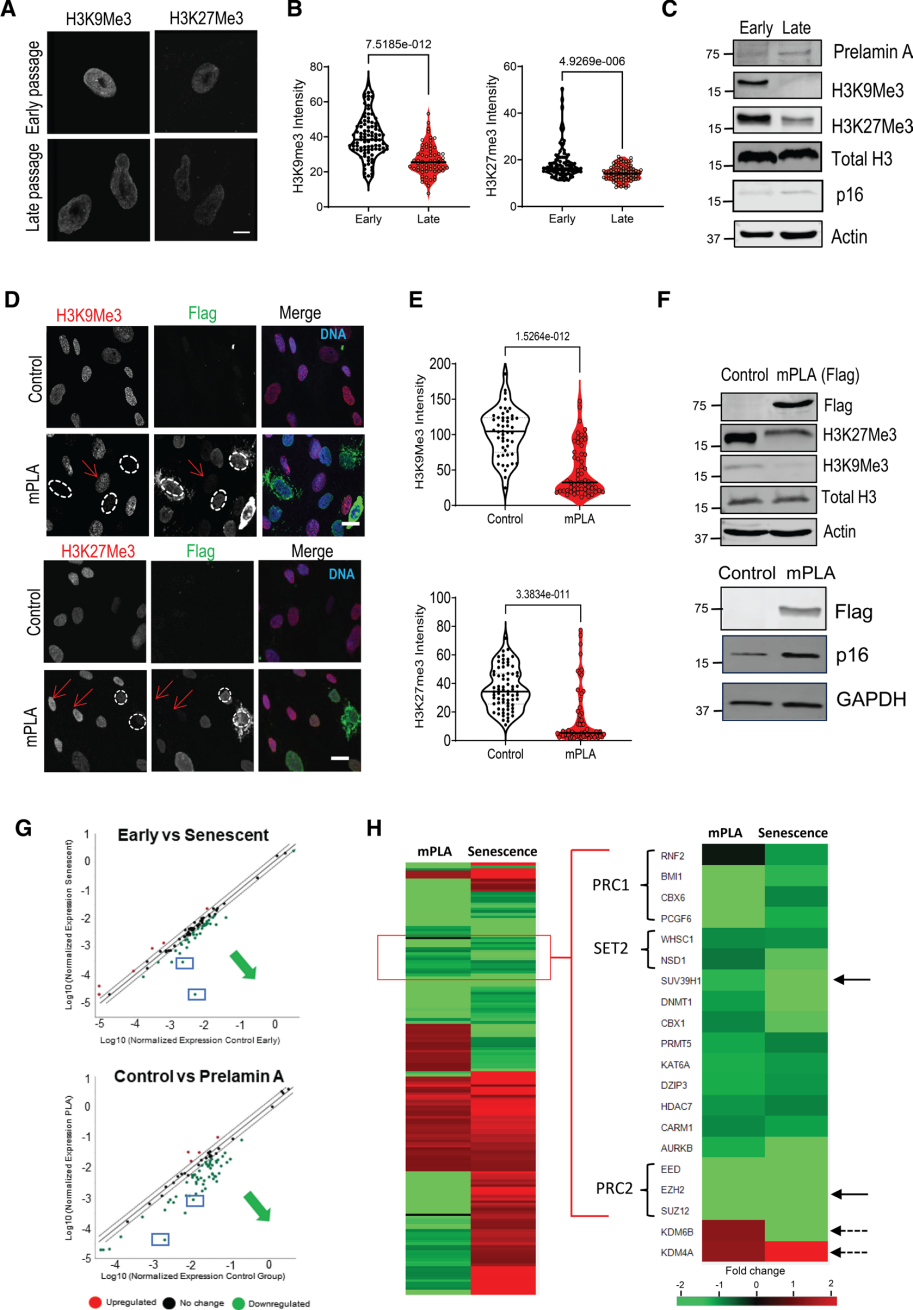
**PLA (prelamin A) accumulation in vascular smooth muscle cells (VSMCs) results in reduction in heterochromatin marks H3K9me3 (histone H3, lysine 9, trimethylation) and H3K27me3 (histone H3, lysine 27, trimethylation) and epigenetic reprogramming. A**, Representative immunofluorescence images of young (passage 4) and senescent (passage 25) VSMCs labeled with anti-H3K9me3 or anti-H3K27me3 antibodies. Scale bar, 10 µm. n=3 independent experiments from 2 biological replicates. **B**, Quantification of nuclear H3K9me3 and H3K27me3 intensity in young and senescent cells. Normality was confirmed for H3K9me3 with the Shapiro-Wilk test; unpaired *t* test and *P* value. Normality was rejected for H3K27me3; Mann-Whitney *U* test and *P* value. **C**, Western blot showed prelamin A expression and decrease of H3K9me3 and H3K27me3 levels and increase of p16 in senescent cells. **D**, Representative immunofluorescence images of VSMCs treated with adenoviral mPLA (prelamin A) or adenoviralEGFP (enhanced green fluorescent protein) control before processing for indirect immunofluorescence using anti-H3K9me3 or anti-H3K27me3 antibodies in conjunction with anti-Flag antibodies and DAPI (4′,6-diamidino-2-phenylindole) counterstain. n=3 independent experiments from 3 biological replicates. Scale bar 20 µm. **E**, Quantification of the nuclear intensity of H3K9me3 and H3K27me3 in control vs mPLA-treated VSMCs showed that prelamin A reduced nuclear levels of H3K9me3 and H3K27me3. n=3 independent experiments in 2 biological replicates. Normality was rejected with Shapiro-Wilk test; Mann-Whitney *U* test and *P* value. **F**, Western blotting showed reduction of H3K9me3 and H3K27me3 and increased p16 protein levels upon PLA expression. **G**, Gene profiling arrays show downregulation of epigenetic regulators upon mPLA expression, which overlaps with the gene expression pattern in senescent VSMCs. Boxes indicate position of SUV39H1 (suppressor of variegation 3–9 homolog 1) and EZH2 (enhancer of zeste 2 PRC polycomb repressive complex 2 subunit). Black line indicates unchanged expression and dashed lines indicate a cutoff of 1.4-fold change above and below the threshold. **H**, Heat maps showing gene expression profiles of epigenetic regulators in senescent and mPLA-expressing VSMCs highlighting changes in heterochromatin modifiers (arrowed). PRC1 indicates polycomb repressive complex1; PRC2, polycomb repressive complex 2; and SET2, SET domain–containing 2, histone lysine methyltransferase.

Primary human VSMCs were grown to replicative senescence by serial passaging in vitro and showed increased expression of p16 as previously described.^12,13^ Senescent cells accumulated PLA at the nuclear envelop and acquired nuclear morphology defects (Figure S1A and S1B). Senescence was associated with a marked reduction of the repressive histone marks H3K9me3 and H3K27me3 (Figure 1A and 1C). To investigate whether the accumulation of PLA promoted these chromatin changes an uncleavable mutant form of PLA (mPLA) was expressed in early passage VSMCs using adenoviral transfection. This led to decreased expression of smooth muscle markers and key cell cycle regulators indicating phenotypic switching and cell cycle arrest (data not shown). A significant reduction in the nuclear intensity of both repressive chromatin marks and increased p16 expression was found after 72 hours post-transfection (Figure 1D and 1F). A similar loss of heterochromatin was observed when zinc metallopeptidase STE24 (*Zmpste24*) which encodes the protein FACE1 (CAAX prenyl proteoase 1 homolog) expression was silenced using siRNA (small interfering ribose nucleic acid)^13^ (Figure S2A through S2D). In contrast, adenoviral expression of wild-type lamin A did not perturb levels of these heterochromatin marks (Figure S2E and S2F).

We hypothesized that dysregulation of epigenetic regulators including writers, erasers and readers may be pivotal in driving these global histone modifications. To determine the status of epigenetic regulators, we performed a targeted polymerase chain reaction (PCR) array on early passage versus senescent VSMCs as well as VSMCs expressing control EGFP (enhanced green fluorescent protein) or mPLA. This analysis revealed rapid and extensive downregulation of key regulators of chromatin structure in mPLA-expressing cells with similar reductions in senescent VSMCs including histone methyltransferases, polycomb complex proteins, and chromobox proteins (HP1 [heterochromatin protein 1] homologs; Figure 1G and 1H). Among these, critical factors for the maintenance of H3K9me3 and H3K27me3 repressive histone modifications, SUV39H1 (suppressor of variegation 3–9 homolog 1; H3K9me3) and EZH2 (enhancer of zeste 2 polycomb repressive complex 2 subunit; H3K27me3), were dramatically decreased in both mPLA-expressing and senescent VSMCs, compared with controls (Figure 1H). In contrast, the histone demethylase KDM4A (lysine demethylase 4A), which regulates H3K9me3, was elevated in mPLA-expressing and senescent VSMCs. The H3K27me3-specific demethylase KDM6B (lysine demethylase 6B), was increased upon mPLA expression but decreased in senescent cells (Figure 1H). Taken together these data suggest that mPLA expression can rapidly recapitulate key aspects of the chromatin environment associated with VSMC senescence.

### Vascular Calcification In Vivo Is Accompanied by Dysregulation of Epigenetic Writers and Erasers Necessary for Maintenance of Repressive Chromatin

**Figure 2.**
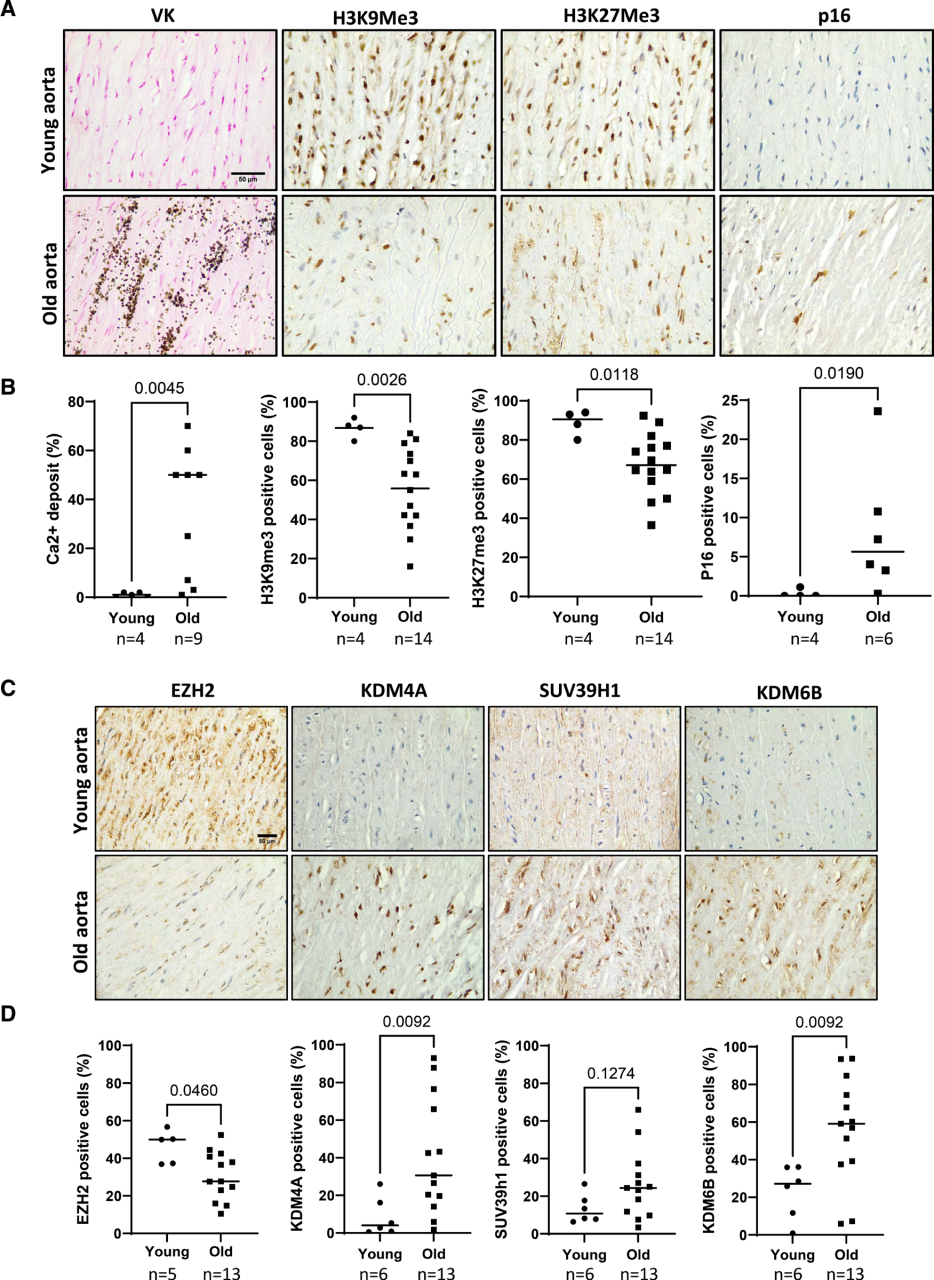
**Aged and chronic kidney disease (CKD) vessels show decreased H3K9me3 (histone H3, lysine 9, trimethylation) and H3K27me3 (histone H3, lysine 27, trimethylation) and perturbed expression of epigenetic regulators. A**, Representative immunohistochemical images of normal and calcified aortic sections stained for H3K9me3, H3K27me3, and p16, showing reduction in nuclei positive for H3K9me3 and H3K27me3 in aged specimens, concurrent with positive labeling for p16. Calcium deposits in aged aorta sections labeled with von Kossa (VK) staining. Scale bar 50 µm. **B**, Quantification of calcium deposition detected by VK staining percent of cells positive for nuclear H3K9me3, H3K27me3, and p16. Significance was analyzed by Mann-Whitney *U* test, and *P* values are shown. **C**, Representative immunohistological images of aortic sections stained for histone methyltransferases SUV39H1 (suppressor of variegation 3–9 homolog 1) and EZH2 (enhancer of zeste 2 polycomb repressive complex 2 subunit) and histone demethylases KDM (lysine demethylase) 4A and 6B. Aged specimens showed reduction in nuclei positive for EZH2, concurrent with increase in nuclei positive for KDM4A and KDM6B. Scale bar 50 µm. **D**, Quantification of percent nuclei positive for KDM4A, KDM6B, SUV39H1, and EZH2. Significance was analyzed by the Mann-Whitney *U* test and *P* values are shown. n indicates numbers as indicated.

PLA accumulation is prevalent in aged and calcified arteries.^12^ Therefore, we next examined whether loss of these repressive heterochromatin marks was also a feature of VSMCs in aged and calcified human arteries in vivo. Immunohistochemistry revealed that VSMCs in calcified aortic samples from older donors (>45 years of age) showed a prominent loss of both H3K9me3 and H3K27me3 marks, compared with vessels from noncalcified young donors (<25 years of age; Figure 2A and 2B). Consistent with the loss of repressive H3K9me3 and H3K27me3 histone modifications in aged arteries, we also observed a coincident decrease in protein expression of the histone methyltransferase EZH2 and a corresponding increase in expression of the histone demethylases KDM4A and KDM6B (Figure 2C and 2D). Loss of heterochromatin coincided with increased p16 staining (Figure 2A and 2B),^15^ suggesting epigenetic alterations coincide with or precede the onset of PLA accumulation and senescence and may act to prime the vessel for subsequent calcification.

### PLA (prelamin A) Overexpression Induced a Shift in Heterochromatin Organization Resulting in Aberrant Activation and Suppression of Genes Associated With Aging and Inflammation

To understand the impact of PLA accumulation in VSMCs and to identify potentially derepressed gene targets that might promote or prime VSMCs for senescence and calcification, we next globally mapped the distribution of H3K9me3 and H3K27me3 patterns across the genome. Chromatin immunoprecipitation (ChIP) followed by sequencing (ChIP-Seq) analysis was performed in VSMCs overexpressing mPLA versus an EGFP control. Most peaks fell in intergenic regions (64.2% in EGFP versus 76.7% in mPLA H3K9me3 ChIP, 61.5% in EGFP versus 58.7% in mPLA H3K27me3 ChIP; Figure S3), consistent with reports that repressive marks are most prominent in noncoding regions and silenced genes. Gene Ontology Biological Process analysis revealed that peaks identified with mPLA expression were associated with DNA damage repair and chromatin maintenance (Figure S3, red boxes) consistent with previous microarray data showing these pathways are downregulated by mPLA.^13^ In addition, consistent with the activation of p16 in response to PLA, visualization of the chromatin landscape revealed reduced H3K9me3 and H3K27me3 peaks around the regulatory domain and promoter regions of the *CDKN2A* gene (Figure S4A). ChIP-qPCR (chromatin immunoprecipitation–quantitative polymerase chain reaction) confirmed that overexpression of mPLA reduced levels of H3K9me3 and H3K27me3 on the *CDKN2A*/p16 promoter (Figure S4B).

**Figure 3.**
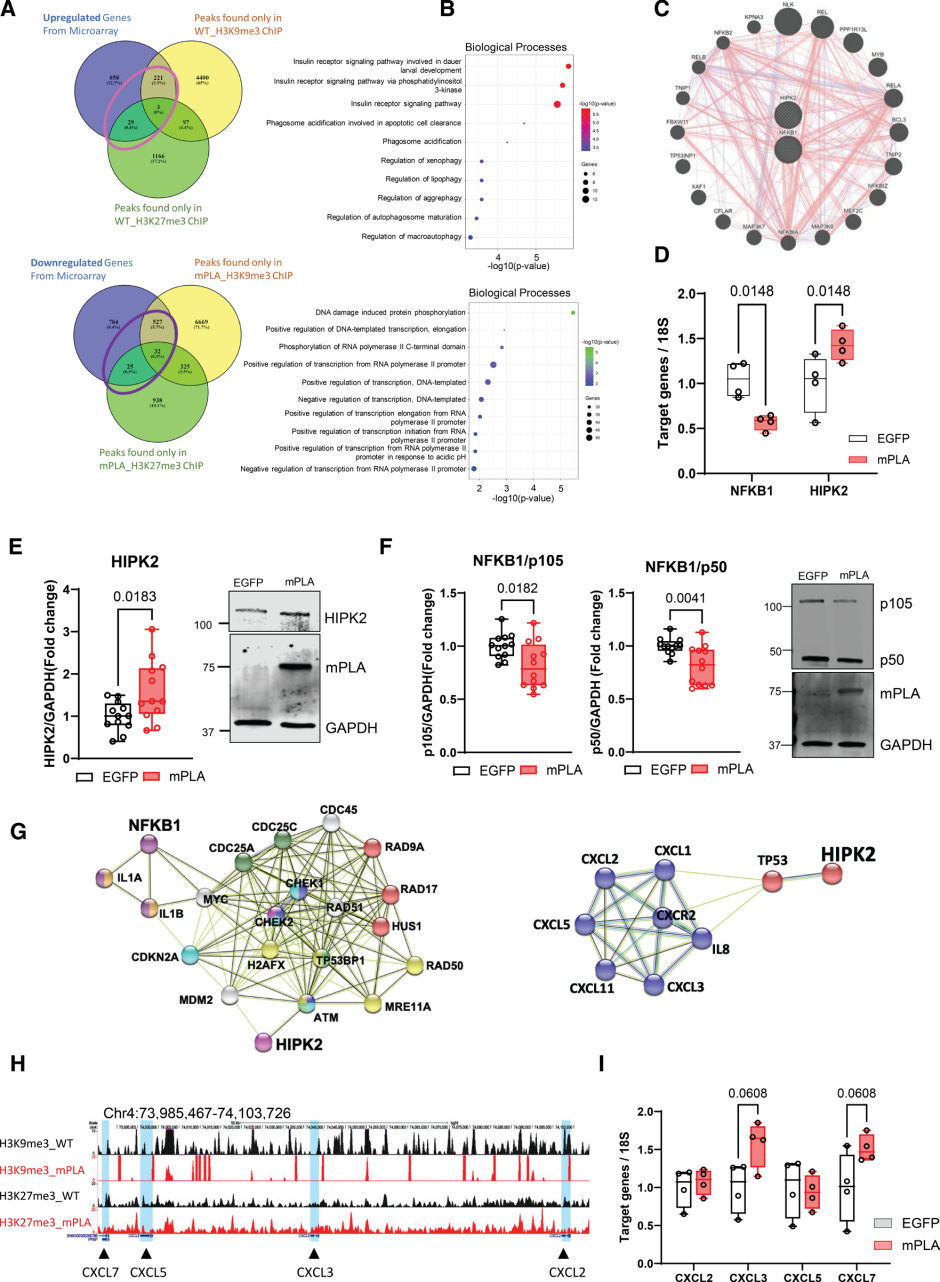
**Chromatin immunoprecipitation followed by sequencing (ChIP-Seq) analysis reveals epigenetic derepression on potential mPLA (prelamin A) targets. A**, **top**, Overlap between upregulated genes in microarray analysis and ChIP-gene annotations found exclusively in wild-type (WT) samples indicate 253 potential gene targets derepressed after reduction in H3K9me3 (histone H3, lysine 9, trimethylation) and H3K27me3 (histone H3, lysine 27, trimethylation) modifications. **Bottom**, Overlap between downregulated genes in microarray analysis and ChIP-gene annotations found exclusively in mPLA samples indicate 584 potential gene targets repressed after reduction in H3K9me3 and H3K27me3 histone modifications. **B**, Gene Ontology analysis of upregulated (253) and downregulated (584) gene targets revealed associations with altered insulin receptor signaling and DNA-binding activities, as top-scoring terms. **C**, Gene network analyses showing homeodomain-interacting protein kinase 2 (*HIPK2*) crosstalk with p53 and NF-κB (nuclear factor κB) signaling pathways. **D**, Nuclear factor of kappa light polypeptide gene enhancer in B cells 1 (*NFKB1*) and *HIPK2* gene expression validation by quantitative real-time polymerase chain reaction (RT-PCR; n=4 from 3 isolates). Mixed model analysis and *q* values adjusted for multiple testing with Benjamini, Krieger, and Yekutieli false discovery rate (FDR) correction are shown. **E**, Western blot showed HIPK2 protein expression was increased in mPLA-expressing samples. n=12 biological repeats from 3 isolates. Normality was confirmed with the Shapiro-Wilk test; unpaired *t* test and *P* value shown. **F**, NFKB1 protein (precursor 105 kDa transcription inhibitor [p105] and cleaved from p50) was decreased in mPLA-expressing samples. n=12 biological repeats from 3 isolates. Normality was confirmed with the Shapiro-Wilk test; unpaired *t* test and *P* value shown. **G**, *HIPK2,* and *NFKB1* regulate DNA damage repair signaling, senescence, and inflammation by String network. **H**, ChIP-Seq analysis showed that repressive histone marks were reduced at the gene loci encoding senescence-associated secretory phenotype proteins. **I**, RT-PCR showing increased expression of growth-related oncogene (*GRO*) locus genes in response to mPLA. n=4 biological repeats from 2 isolates. Mixed model analysis and *q* values adjusted for multiple testing with Benjamini, Krieger, and Yekutieli FDR correction are shown. ATM indicates ataxia-telangiectasia mutated; CDC, cell division cycle; CDKN, cyclin dependent kinase inhibitor; CHEK, checkpoint kinase; CXCL, C-X-C motif chemokine ligand; EGFP, enhanced green fluorescent protein; H2AFX, H2A.X variant histone; HUS, checkpoint clamp component; IL, interleukin; MDM, protoncogene, E3 ubiquitin protein ligase; MRE, double strand break repair nuclease; MYC, myc protooncogene, BHLH transcription factor; RAD, checkpoint clamp component; TP53, tumor protein P53; and TP53BP, tumor protein P53 binding protein.

Next, using an unbiased approach to identify biologically relevant gene targets modified by mPLA-induced changes in the epigenetic landscape, we analyzed the ChIP-seq dataset against published microarray expression data of control EGFP and mPLA-expressing VSMCs.^13^ Further screening using criteria of upregulation in microarray analysis (cut off *P*=0.05) after mPLA expression and displaying binding peaks within ±5 kb from the transcription start site present exclusively in the control ChIP, we identified 253 genes that are potentially derepressed by loss of heterochromatin as a result of mPLA expression (Figure 3A). Gene Ontology biological processes identified genes involved in metabolic pathways especially insulin signaling and regulation of autophagy (Figure 3B). Among the 3 elements that fulfilled all criteria, homeodomain-interacting protein kinase 2 (*HIPK2*), a key player involved in connecting p53/TP53 (tumor protein p53)–mediated cell cycle events and modulating Wnt (wingless-type MMTV integration site family member)/beta-catenin signaling was identified.^34^ Conversely, when we applied filters for genes that were downregulated upon mPLA expression and binding peaks within ±5 kb from the transcription start site present exclusively in the mPLA ChIP, we observed that the top hits were genes involved in transcriptional regulation and DNA damage repair pathways (Figure 3A and 3B). Of the 32 genes that met all criteria, the nuclear factor of kappa light polypeptide gene enhancer in B cells 1 (*NFKB1*) which can also crosstalk with HIPK2 was revealed as a downregulated target that could be subjected to epigenetic suppression (Figure 3C). This gene encodes a 105 kDa transcription inhibitor (p105) which can be processed to yield the 50 kDa DNA-binding subunit of the NF-κB (nuclear factor κB) protein complex. The p105 precursor is involved in a variety of NF-κB independent functions while p50 homodimers suppress the NF-κB response.^35^ Quantitative real-time PCR (qRT-PCR) and Western blot analysis verified expression changes for both genes as well as changes in protein levels for HIPK2 and NFKB1 subunits (Figure 3D and 3F). Gene network analysis showed HIPK2 and NFKB1 are closely linked with DNA damage repair pathways, senescence, and chemokine signaling and can crosstalk to regulate NF-κB signaling a key mediator of the inflammatory SASP response (Figure 3G).^36^

As robust SASP activation is a major outcome of mPLA expression in VSMCs, we also examined the chromatin landscape around clustered SASP genes previously shown to be actively secreted by mPLA-expressing VSMCs.^13^ Examination of the peaks associated with the growth-related oncogene (*GRO*) locus on chromosome 4 (Figure 3H) showed a marked reduction in H3K9me3 and a modest reduction in H3K27me3 in association with C-X-C motif chemokine ligand (*CXCL*) *2, 3, 5* and 7 compared with EGFP control. qRT-PCR confirmed that members of this cluster, including *CXCL3* and 7 were upregulated in response to mPLA, suggesting that large-scale alterations in the epigenetic landscape can potentially lead to coordinated activation of SASP gene clusters (Figure 3I). This derepression of chemokine expression together with, or in response to, deregulated NF-κB and DNA damage signaling likely contribute to the robust inflammatory SASP phenotype displayed by mPLA-expressing VSMCs.^13^

### Smooth Muscle-Specific PLA (prelamin A) Expression Induces Heterochromatin Loss and Changes in Gene Expression Patterns

To study further the temporal relationship between loss of heterochromatin and gene activation in vivo, we designed a tamoxifen-inducible, VSMC-specific PLA mouse model (mPLA/Cre^ERT2^) by crossing transgenic mice with a mPLA transgene with a floxed stop codon with mice harboring a CreER^T2^ transgene^37^ where expression of Cre recombinase with the modified estrogen receptor binding domain was driven by the *MYH11* promoter to restrict expression to VSMCs.^38^

**Figure 4.**
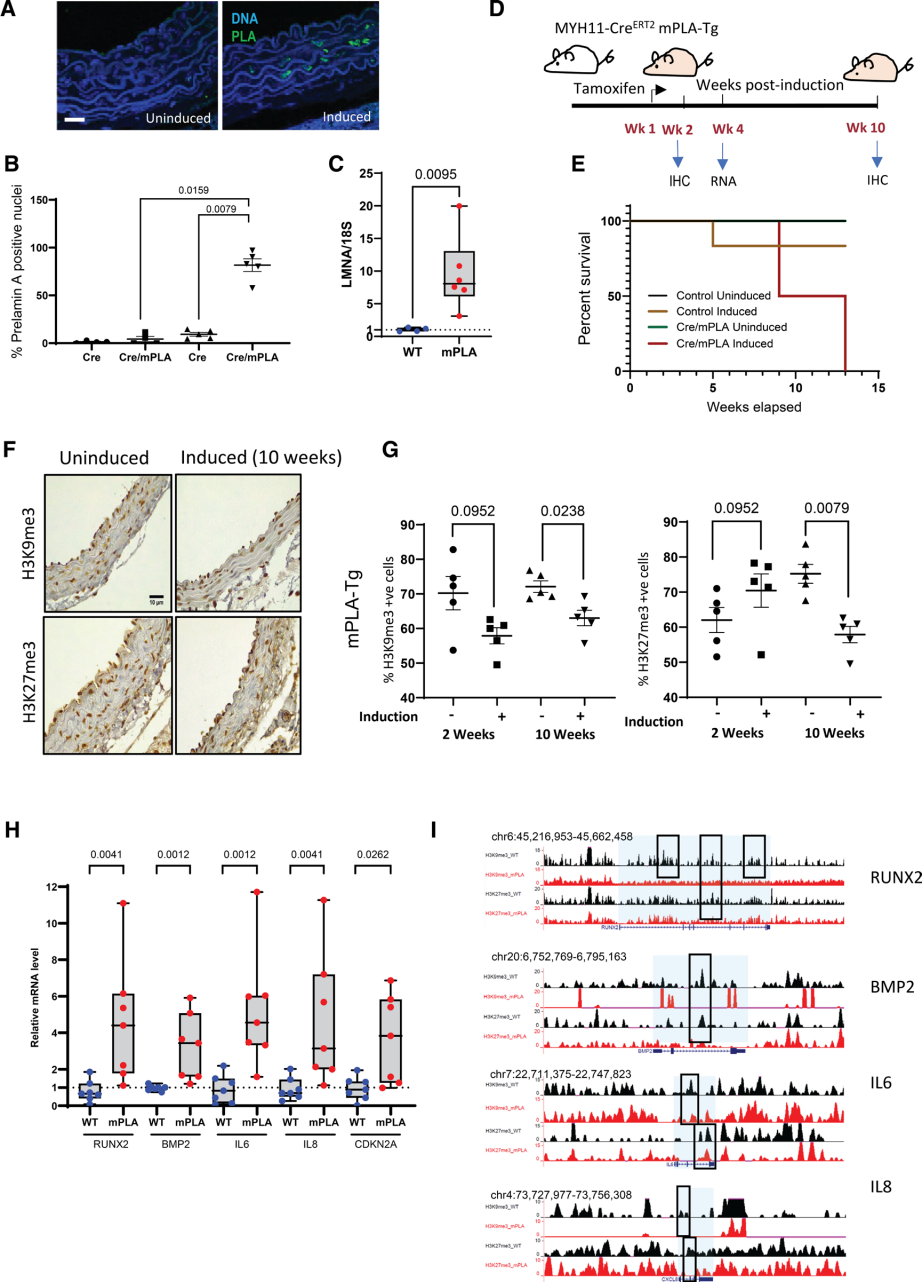
**Vascular smooth muscle cell (VSMC)–specific mPLA (prelamin A) expression in vivo results in early reduction in heterochromatin marks. A** and **B**, Immunofluorescence showing induction of PLA expression in VSMCs upon tamoxifen treatment (2 weeks). **B**, PLA was significantly increased upon tamoxifen treatment. N=4 in the uninduced group and n=5 in the tamoxifen-induced group. Mann-Whitney *U* test *P* values are shown. **C**, Lamin A/C (*LMNA*) transcript was increased significantly in the tamoxifen-induced group (mPLA). N=6. Mann-Whitney *U* test *P* values are shown. **D**, Experimental design of tamoxifen induction of mPLA-transgenic (Tg) mice or control littermates. **E**, Survival of uninduced and induced mPLA-Tg mice (control n=14 and mPLA-Tg n=6). **F**, Representative immunostaining of H3K9me3 (histone H3, lysine 9, trimethylation) and H3K27me3 (histone H3, lysine 27, trimethylation) in aortas from uninduced and induced mPLA-Tg mice at 2 weeks. Scale bar, 10 mm. n=5 mice per group. **G**, Quantification of H3K9me3 and H3K27me3 in aortas from uninduced and induced mPLA-Tg mice at 2 and 10 weeks. Normality confirmed with Shapiro-Wilk test; Mann-Whitney *U* test *P* values are shown. **H**, Quantitative real-time polymerase chain reaction showing increased expression of osteogenic genes, senescence-associated secretory phenotype genes, and cyclin dependent kinase inhibitor 2A (*CDKN2A*) in mPLA-Tg expressing mice (4 weeks). N=7 mice per group. Mann-Whitney *U* test *P* values are shown. **I**, chromatin immunoprecipitation followed by sequencing analysis showed that repressive histone marks were reduced at the gene loci encoding genes upregulated by PLA expression in **H**. BMP 2 indicates bone morphogenetic protein 2; IHC, immunohistochemistry; IL, interleukin; Runx2, runt-related transcription factor 2; Tg, transgenic; and WT, wild-type.

Tamoxifen treatment resulted in increased expression of *LMNA* from the transgene and ≈80% of vessel VSMCs accumulating PLA protein (Figure 4A and 4D) 2 weeks post-tamoxifen induction. Expression of PLA in 8- to 10-week-old mice resulted in a sharp decline in lifespan and healthspan of the transgenic mice accompanied by stunted growth and death at around 12 weeks (Figure 4E; Figure S5A). PLA–positive aortas collected at 2 and 10 weeks post-induction showed vessels were generally normal with no changes in normalized cell density, collagen, or glycosaminoglycan content and mineralization was not present. Some perivascular fibrosis in coronary arteries was observed at 10 weeks; however, the cause of death remained undefined (Figure S5B through S5E).

Significant depletion in the repressive histone marks H3K9me3 and H3K27me3 was observed at the 10-week time point (Figure 4F and 4G). At this time point, significantly increased expression of the senescence marker p16 or the DNA damage marker γH2AX (gamma histone variant H2AX) was not observed consistent with epigenetic changes preceding and priming the vessel wall for pathological change (Figure S5F and S5G).

We were interested to know if loss of heterochromatin was associated with early expression of senescence, osteogenic, or inflammatory genes previously shown to be increased by PLA in vitro. Aortas were harvested from mice at 4 weeks post-tamoxifen induction, and qRT-PCR was performed. Strikingly, we observed upregulation of *CDKN2A*, runt-related transcription factor 2 (*RUNX2*), and the osteogenic SASP factors *BMP2*, interleukin 6 (*IL6*), and *IL8*, suggesting that these genes are rapidly derepressed in response to heterochromatin loss in vivo (Figure 4H). Indeed, cross-referencing to the epigenetic landscape of these genes in the in vitro human PLA ChIP-seq dataset showed a dramatic loss of peaks of both H3K9me3 and H3K27me3 on these loci (Figure 4I).

**Figure 5.**
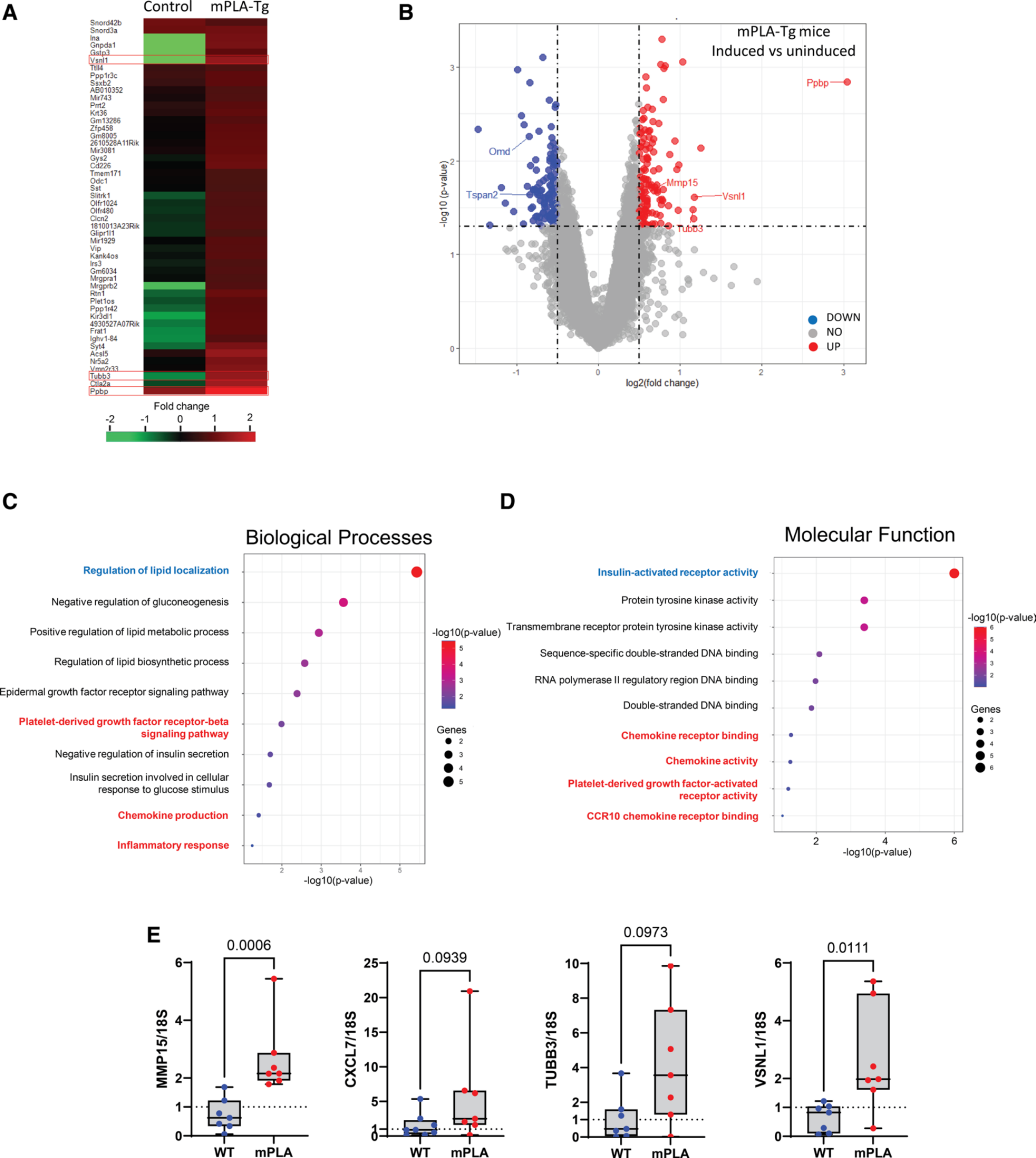
**Mice expressing mPLA (prelamin A) show early induction of chemokine signaling and accelerated calcification. A**, Heat map showing differentially expressed genes in mPLA-Tg expressing mice vs uninduced controls shown as fold change. **B**, Volcano plot showing significantly upregulated and downregulated genes. Cut off: log2FC=0.5 with *P*=0.05. Indicated genes have previously been shown to have a role in vascular calcification or were selected for validation by quantitative real-time polymerase chain reaction (qRT-PCR). **C** and **D**, Gene Ontology analysis pathways significantly altered in response to PLA expression. **E**, qRT-PCR verification of differential gene expression in mouse aorta expressing mPLA-transgenic (Tg). n=7 mice per group. Mann-Whitney *U* test *P* value shown. CCR indicates C-C motif chemokine receptor; CXCL7, C-X-C motif chemokine ligand 7; MMP, matrix metalloproteinase; TUBB3, tubulin beta 3; VSNL1, visinin-like 1; and WT, wild-type.

We next performed microarray analysis to determine the global gene expression changes induced by loss of heterochromatin in vivo. Approximately 1000 differentially expressed genes were obtained at *P*<0.05 induced only in mPLA-transgenic (Tg) mice (Figure 5A and 5B). Gene Ontology Biological Processes and Molecular Function analyses revealed associations with altered metabolic signaling including lipid and glucose metabolism and insulin signaling as well as DNA damage repair. There was also an association with inflammatory responses including chemokine activity and signaling. Negative regulation of PDGF (platelet-derived growth factor) signaling, which is associated with phenotypic modulation of VSMCs, was also among the most affected processes (Figure 5C and 5D). Volcano plot analysis showed that *Ppbp/Cxcl7* which belongs to the GRO cluster, in close proximity to the *Cxcl2*, *Cxcl3*, and *Cxcl5 g*enes on chromosome 5 was among the top hits for upregulated genes upon PLA induction (Figure 5B). Among the other genes whose expression was increased were visinin-like 1 *(Vsnl1*), a calcium-sensing protein, that has been associated with coronary artery calcified atherosclerotic plaque in a type 2 diabetes genome-wide association study,^39^ and matrix metalloproteinase 15 (*Mmp15*) a regulator of extracellular matrix remodeling.^40^ Tubb3 (tubulin beta 3) has been implicated in TGFβ (transforming growth factor beta)−signaling, fibrosis, and endothelial-mesenchymal transition.^41^ Of the downregulated gene targets the tetraspanin 2 (*Tspan2*) gene is a key regulator of VSMC differentiation^42^ and lies close to a large artery ischemic stroke susceptibility locus associated with intraplaque calcification,^43^ whereas Omd (osteomodulin) has recently been identified as a biomarker for vascular calcification.^44^ qRT-PCR was used to verify the changes in the identified genes (Figure 5E).

### VSMC Calcification Induced by Mineral Dysregulation Is Associated With Rapid Loss of Repressive Histone Modifications and Early Induction of Inflammation

**Figure 6.**
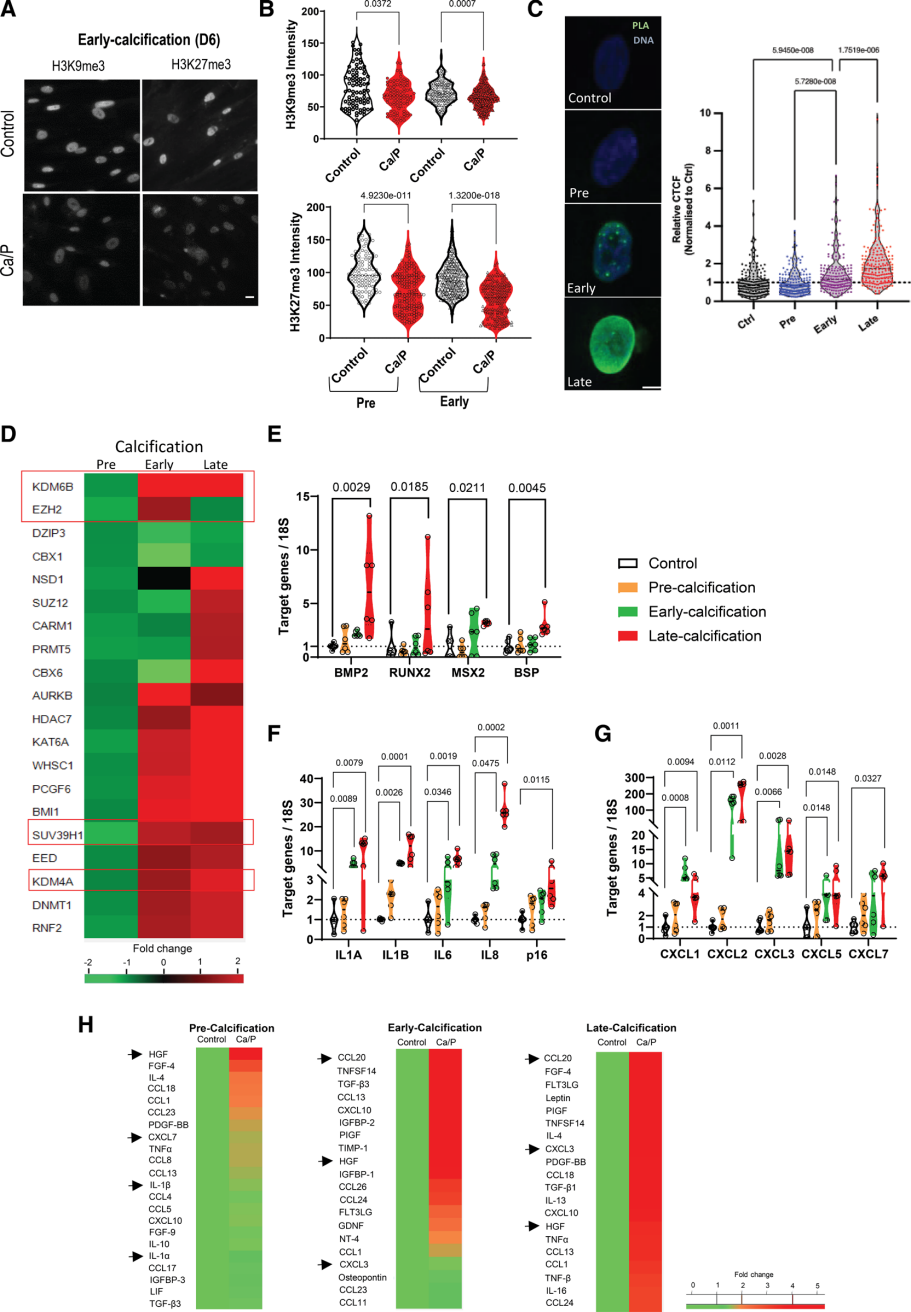
**Vascular smooth muscle cells (VSMCs) display early senescence-associated secretory phenotype (SASP) activation during calcification, which is correlated to reduction in H3K9me3 (histone H3, lysine 9, trimethylation) and H3K27me3 (histone H3, lysine 27, trimethylation) modifications. A**, VSMCs showed reduction in H2K9me3 and H3K27me3 during early calcification. Scale bar 10 µm. **B**, Quantification of nuclear intensities of H3K9me3 and H3K27me3 immunostaining in VSMCs treated with control media (Control) or calcifying calcium and phosphate (Ca/P) media. n=3 independent experiments from 3 isolates. Kruskal-Wallis test and *P* value are shown. **C**, Increasing PLA expression during VSMC calcification. Quantification of nuclear intensities of PLA immunostaining in VSMCs at the different stages of calcification shown as relative calculated corrected total cell fluorescence (CTCF). n=4 independent experiments from 2 isolates. Kruskal-Wallis test and *P* value are shown. **D**, Heat map of expression changes of selected epigenetic regulators from polymerase chain reaction array over the time course of calcification. H3K9m3 and H3K27me3 regulators are boxed. SUV39H1 (suppressor of variegation 3-9 homologue 1), EZH2 (enhancer of zeste 2 homologue 2), KDM (lysine demethylase) 6B and 4A. **E**, In response to calcifying media VSMCs upregulate osteogenic markers at the late calcification stage. n=6 independent experiments from 3 biological replicates. Kruskal-Wallis test and *q* values adjusted for multiple testing with Benjamini, Krieger, and Yekutieli false discovery rate (FDR) correction are shown. **F** and **G**, Robust SASP activation occurs at the early calcification stage. n=6 independent experiments from 3 biological replicates. Kruskal-Wallis test and *q* values adjusted for multiple testing with Benjamini, Krieger, and Yekutieli FDR correction are shown. **H**, Conditioned media from VSMCs treated with calcifying calcium and phosphate (Ca/P) media were harvested at each stage of calcification for antibody cytokine array analysis. Densitometry was performed and normalized to control conditions. Heat maps show fold changes for the top 20 upregulated cytokines from each stage of calcification compared to control for each timepoint. n=2 for precalcification, n=1 for early calcification and n=2 for late calcification. Arrows indicate abundant cytokines released across the timecourse. Arrows indicate factors increased across the timecourse and implicated in calcification. BSP indicates integrin-binding sialoprotein; CCL CC chemokine ligand; CXCL, BMP bone morphogenetic protein; C-X-C motif chemokine ligand; FGF, fibroblast growth factor; HGF, hepatocyte growth factor; IGFBP, insulin like growth factor binding protein; IL, interleukin; LIF, leukemia inhibitory factor; Msx2, Msh homeobox 2; NT, neurotrophin; PDGF, platelet derived growth factor; PIGF, phosphatidylinositol glycan anchor biosynthesis class F; Runx2, runt-related transcription factor 2; TGF, transforming growth factor; TIMP, tissue inhibitor of metalloproteinase; and TNF, tumor necrosis factor.

Mineral dysregulation is a key driver of vascular aging and calcification.^6,45^ To understand if epigenetic perturbation is an early feature of mineral stress–induced calcification VSMCs were cultured in vitro in osteogenic media containing elevated levels of calcium and phosphate to model the mineral disturbances observed in CKD.^13^ Three stages were defined as precalcification, day 2 (no mineralization), early calcification, day 6 (microcalcification visible) and late calcification, and day 9 (extensive deposition of mineral; Figure S6A and S6B). Immunofluorescence showed that in response to osteogenic media both H3K9me3 and H3K27me3 were reduced during the earliest phase of treatment (day 2) and further reduced with the onset of calcification (day 6), indicating loss of these marks precedes calcification (Figure 6A and 6B). H3K27me3 showed the greatest reduction in response to mineral dysregulation, and this was confirmed by Western blot at the late calcification time point (Figure 6B; Figure S6C and S6D). In parallel with heterochromatin loss, IF showed significant nuclear accumulation of PLA at the early time point with levels rising still further as calcification progressed (Figure 6C).

To confirm whether reductions of repressive histone marks were related to changes in histone modifiers, we again performed targeted transcriptome analysis of epigenetic regulators and found that the transcriptional profile of these regulators displayed dynamic changes. At the initial phase (day 2), VSMCs exposed to osteogenic media showed a similar profile to PLA expressing and senescent VSMC populations, with both SUV39H1 and EZH2 downregulated, consistent with the early reduction in histone modifications observed (Figure 6D). As calcification progressed (day 6–9), the transcriptional profile indicated extensive upregulation of DNA and histone modifying enzymes and chromatin modifiers, including KDM4A/6B, suggesting a global, rewriting of the histone code, giving rise to an epigenetic landscape that is unique to calcified VSMCs in vitro (Figure 6D).

We next used qRT-PCR analysis to determine how the temporal pattern of gene expression for osteogenic, SASP, and senescence markers correlated with the loss of heterochromatin marks and onset of calcification. VSMC calcification was accompanied by increased gene expression of the senescence marker p16 as well as osteogenic markers Msx2 (Msh homeobox 2), Runx2, and its downstream target BSP (integrin-binding sialoprotein). However significant upregulation of these markers occurred late in the calcification process (day 9; Figure 6E). In contrast, examination of abundant inflammatory SASP factors released by VSMCs showed that transcription was significantly increased during the early phase of calcification (day 6; Figure 6F). Upregulated SASP genes included those previously shown to be activated in response to PLA including the osteogenic factors IL6, BMP2, and IL-1α and IL-1β, as well as coordinate increases in clustered chemokines mapping to the *GRO* locus, including CXCL1, CXCL2, CXCL3, CXCL5, CXCL7, and CXCL8 (IL8; Figure 6F and 6G).^13^

Cytokine antibody array analysis over the time course of calcification (Figure 6H) showed that as early as the precalcification stage (day 2), coinciding with the loss of heterochromatin marks, secretion of abundant SASP factors was markedly increased. Again, these included factors previously reported to be induced by PLA, such as IL-1α and IL1β, CCL20 (C-C motif chemokine ligand 20) , hepatocyte growth factor (HGF), and CXCL3, with both IL-1β and HGF increases occurring precalcification and persisting into late calcification (day 9; Figure 6H).^13^ The GRO cluster chemokine CXCL7, and members of the clustered CCL1/CCL18 (MCP [monocyte chemotactic protein] region) and CCL18/CCL23 (MIP [macrophage inflammatory protein] region; Figure 6H; Figure S7), were also increased. Taken together, these data confirm that inflammation precedes osteogenic differentiation and senescence and that osteogenic stimuli and aging drive a similar inflammatory SASP.

### Inflammation Precedes VSMC Calcification and Is Exacerbated by Inhibition of SUV39H1 and EZH2

**Figure 7.**
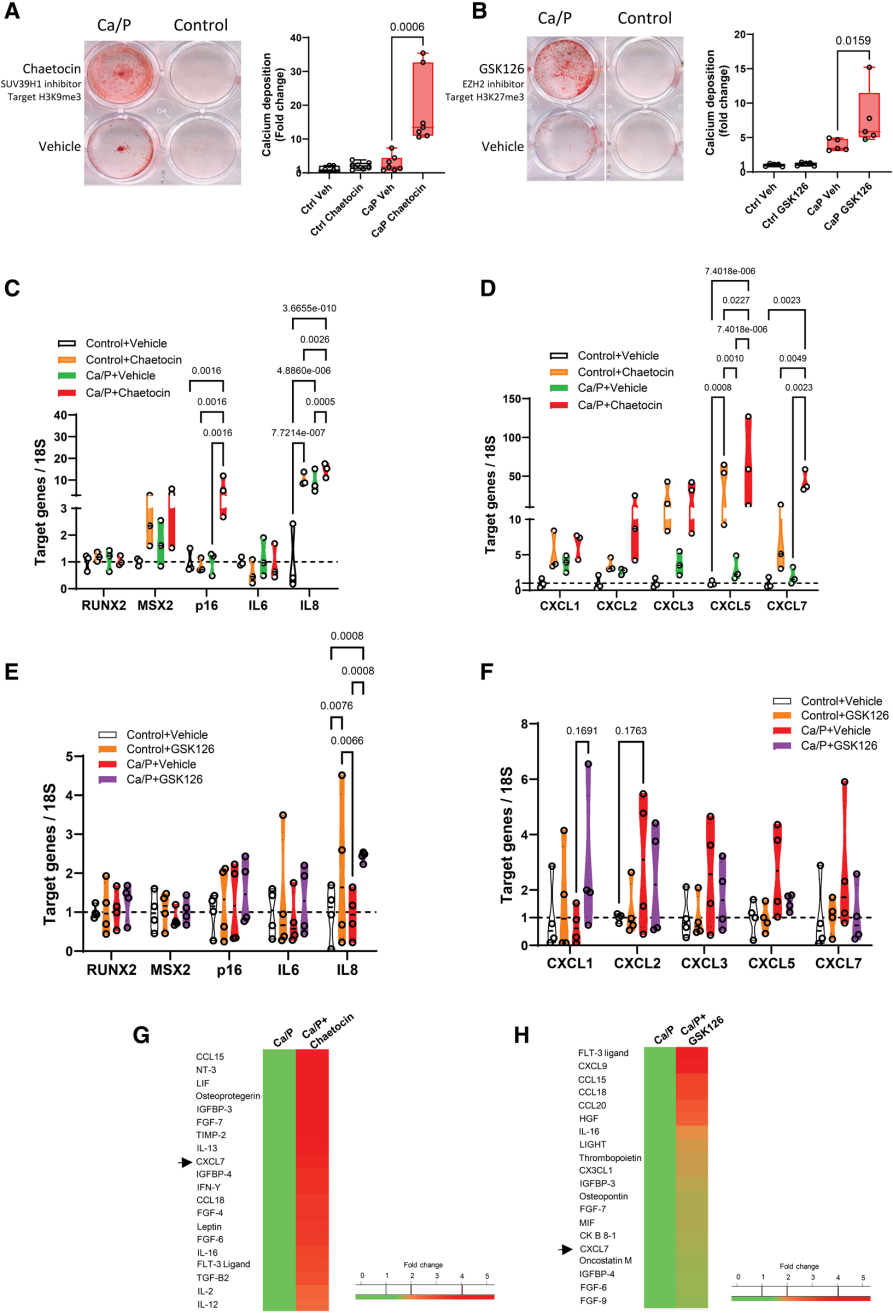
**SUV39H1 (suppressor of variegation 3–9 homolog 1) and EZH2 (enhancer of zeste 2 polycomb repressive complex 2 subunit) inhibitors accelerate vascular smooth muscle cell (VSMC) calcification in vitro and induce senescence-associated secretory phenotype (SASP) activation. A** and **B**, Representative images of Alizarin Red staining of calcified cells and quantification of calcium accumulation after treatment with chaetocin (**A**) or GSK126 (**B**). Ctrl, control media, Vehicle, dimethyl sulfoxide (DMSO) control. n=3 independent experiments in 2 biological replicates. Significance was analyzed by Mann-Whitney *U* test and *P* values are shown. **C** through **F**, Gene expression changes quantified by quantitative real-time polymerase chain reaction (qRT-PCR; normalized to 18S expression) showed an increase in SASP genes after treatment with EZH2 or SUV39H1 inhibitors under control or calcium and phosphate (Ca/P) conditions. n=3 for Chaetocin treatment and n=4 for GSK126 treatment from 3 biological replicates. Mixed model analysis and *q* values adjusted for multiple testing with Benjamini, Krieger, and Yekutieli false discovery rate (FDR) correction. **G** and **H**, Conditioned media of VSMCs treated with high Ca/P media in the presence or absence of Chaetocin (6 days) and GSK126 (3 days) were collected and used for cytokine antibody arrays. The top 20 upregulated chemokines (2 independent experiments) are presented as heat maps compared with control cells without drug treatment. Arrow denotes upregulated secretion of CXCL7 (C-X-C motif chemokine ligand 7) upon treatment with chaetocin and GSK126. IL indicates interleukin; Msx2, Msh homeobox 2; and Runx2, runt-related transcription factor 2.

To examine the idea that key epigenetic regulators of heterochromatin can establish the SASP and downstream vascular calcification, we next selectively blocked the activities of histone methyltransferases SUV39H1 and EZH2 using the small molecule inhibitors chaetocin and GSK126 to reduce levels of H3K9me3 and H3K27me3 respectively. In control media, there was no induction of calcification; however, the expression of osteogenic markers and p16 were highly elevated in some individual cell isolates (Figure 5A and 5F; Figure S8A through S8D). Expression of IL8 was significantly increased in response to SUV39H1 and EZH2 inhibition, whereas chemokines at the GRO locus were significantly increased in response to SUV39H1 inhibition (Figure 7C and 7F). In osteogenic media, both inhibitors enhanced calcification and this was associated with dramatic enhancement of SASP gene expression and increased expression of p16 particularly in response to SUV39H1 inhibition (Figure 7C and 7D). Consistent with this enhanced gene expression in response to osteogenic media, cytokine array analysis again revealed increased secretion of chemokines including CXCL7 (*GRO* locus) in response to both drugs (Figure 7G and 7H). Treatment with these inhibitors also caused elevation in secretion of growth factors and other inflammatory mediators with important roles in modulating smooth muscle phenotype and vascular calcification, such as osteopontin, osteoprotegerin, members of the TGF superfamily, and IGFBP3 (insulin like growth factor binding protein 3), consistent with loss of heterochromatin perturbing VSMC phenotype^46^ (Figure 7G and 7H).

### PLA Accumulation and Heterochromatin Loss Are Features of CKD-Induced Vascular Calcification

**Figure 8.**
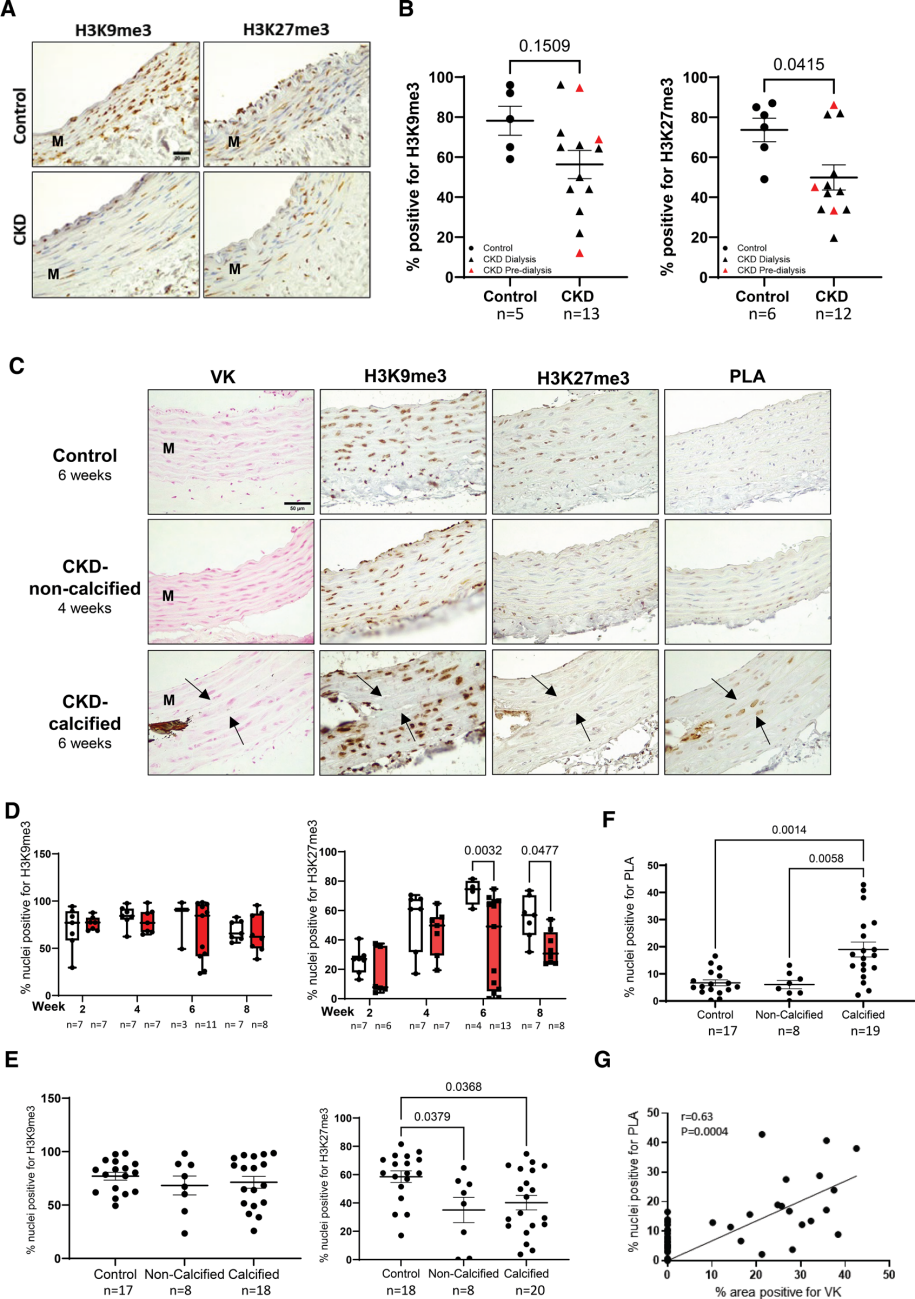
**Reduction of repressive histone marks determined in patients with chronic kidney disease (CKD) and rat CKD model. A**, Representative immunostaining of H3K9me3 (histone H3, lysine 9, trimethylation) and H3K27me3 (histone 3, lysine 27, trimethylation) in aortas from control (non-CKD) and children with CKD. Scale bar=20 µm. **B**, Quantification of percent positive nuclei showed H3K27me3 was reduced in patients with CKD. Red dots in CKD group represent predialysis patients. Significance was analyzed by Mann-Whitney *U* test and *P* value is shown. **C**, Representative images of von Kossa (VK) stain and immunostaining for repressive histone marks and PLA (prelamin A) in calcified and noncalcified aortas from CKD rats. Arrows indicate the same area in serial sections showing PLA accumulation and loss of heterochromatin marks at calcification sites. **D**, Percent positive nuclei for H3K9me3 and H3K27me3 in CKD rats at time points 2, 4, 6, and 8 weeks after exposure to adenine diet showing reduction in H3K27me3 at 6 and 8 weeks. n numbers are indicated. Mixed model analysis and *q* values adjusted for multiple testing with Benjamini, Krieger, and Yekutieli false discovery rate (FDR) correction are shown. **E**, Percent positive nuclei for H3K9me3 and H3K27me3 in control and CKD rats pooled from 4, 6, and 8 weeks comparing calcified and noncalcified aorta. Kruskal-Wallis test and *P* value are shown. **F**, PLA expression in control and CKD rats pooled from 4, 6, and 8 weeks comparing calcified and noncalcified aorta. Kruskal-Wallis test and *P* value are shown. **G**, Spearman correlation analysis of calcification and PLA in pooled CKD rats. M indicates media.

We have previously shown that children on dialysis show accelerated vascular aging with increased levels of p16 and PLA.^13,15^ To understand whether dysregulated mineral metabolism was also associated with accelerated loss of heterochromatin in vivo, we performed IHC. This revealed reductions in the heterochromatin marks with a significant loss of H3K27me3 in vessels from children with CKD (Figure 8A and 8B). Loss of heterochromatin preceded the onset of overt calcification indicated by the absence of von Kossa staining in the majority of vessels from children with CKD either on dialysis or predialysis (Table S3). We also exposed a subset of these vessels ex vivo to osteogenic media for 10 days. Vessels exposed to mineral stress showed a loss of H3K27me3 in the majority of samples with H3K9me3 generally unchanged (Figure S9).

To explore further the time course of heterochromatin loss in response to mineral dysregulation in vivo and its relationship with PLA accumulation and calcification, we used the rat adenine model of CKD. IHC was used to analyze von Kossa, heterochromatin marks, and PLA at weeks 2, 4, 6, and 8 on the adenine diet. Calcification in the rat CKD model is heterogeneous. The percentage of animals showing calcification increased over the time course with no animals calcified at week 2, whereas 3 of 7 (42.85%), 11 of 13 (84.6%), and 6 of 8 (75.0%) were calcified at 4, 6, and 8 weeks, respectively. Although there was no difference between controls and CKD rats at the 2- and 4-week time points, loss of the heterochromatin mark H3K27me3 was observed at weeks 6 and 8, with no change in H3K9me3 (Figure 8C and 8D). Close examination of staining in regions abutting calcification showed that loss of both H3K27me3 and H3K9me3 was observable where PLA accumulation was most abundant but these sites were rare so it was not possible to accurately quantify H3K9me3 loss (Figure 8C). Importantly, pooling the rats into calcified and noncalcified groups showed H3K27me3 was reduced in both groups confirming heterochromatin loss precedes calcification (Figure 8E) In contrast, PLA accumulation was only observed in the calcified rats and strongly correlated with the level of calcification (Figure 8F and 8G). Taken together, these data imply that mineral dysregulation can induce early loss of heterochromatin and is a priming event for calcification. The accumulation of PLA only in calcified arteries suggests it is a key accelerant of calcification acting via multiple mechanisms including induction of heterochromatin loss, DNA damage, inflammation, and osteogenic differentiation.

## Discussion

In this study, we show that loss of heterochromatin is an early and ubiquitous feature of vascular aging, preceding the onset of vascular calcification. We show that prelamin A, a biomarker of vascular aging, acts to rewrite the epigenetic landscape of VSMCs, causing loss of heterochromatin and promoting a proinflammatory senescence-like phenotype mimicking features of senescent cells. VSMCs with reduced heterochromatin show changes in metabolic, homeostatic, and inflammatory pathways linked to aging and were primed to respond to additional stress which caused further robust activation of senescence and SASP pathways and accelerated calcification. Importantly, metabolic stress in VSMCs, caused by dysregulated mineral metabolism was also shown to drive loss of heterochromatin and early and robust activation of SASP pathways suggesting it may be an important environmental factor influencing epigenetic aging. This was supported by data showing loss of heterochromatin before calcification in vessels from children on dialysis and in aortic tissue from rats with CKD. These observations are consistent with emerging evidence implicating mineral metabolism and serum phosphate as key drivers of inflammaging and calcification with nuclear lamina dysfunction and loss of heterochromatin acting to accelerate these processes.^6,40,47^

### PLA and Metabolic Stress Drive Premature Senescence via Loss of Heterochromatin

VSMCs activate a unique senescence program mediated by p16 and characterized by the accumulation of PLA and loss of heterochromatin without senescence-associated heterochromatin foci formation, which are typical of other cell types.^31,48^ Loss of heterochromatin and absence of senescence-associated heterochromatin foci are also epigenetic characteristics of Hutchinson-Gilford Progeria Syndrome cells, occurring in parallel with changes in chromosomal compartmentalization, compaction, and disruption of lamina-associated domains; however, these latter features have not been investigated in VSMCs.^28^ Here, we show that expression of PLA in healthy, proliferating VSMCs can rapidly mimic aspects of the epigenetic landscape of senescent VSMCs with loss of heterochromatin and pronounced depletion of epigenetic writers and readers suggesting similarities in chromatin accessibility and transcriptional profiles with senescent cells. We also documented rapid induction of p16 expression and reduced occupancy of repressive H3K9me3 and H3K27me3 heterochromatin marks along regulatory regions of the *CDKN2A* locus consistent with the current model that loss of heterochromatin derepresses this locus to effect cellular senescence.^33^ Two key histone methyltransferases, SUV39H1 and EZH2, which are dedicated epigenetic writers for H3K9me3 and H3K27me3, respectively, were found to be downregulated, whereas histone demethylases were increased, consistent with the reduction in both repressive histone modifications. Deregulation of expression of these factors was also a feature of aged and calcified human vessels in vivo and could be partially mimicked by mineral stress in vitro. Loss of heterochromatin in vessels from children with CKD where old age as a confounding factor is absent, as well as its loss in a rodent model of CKD, further supports a role for mineral dysregulation in driving heterochromatin loss.^15^

### Epigenetic Modulation of Key Regulators of Inflammation in PLA–Expressing Cells

To study the earliest events leading to senescence and the functional consequences of loss of heterochromatin, we used PLA as a model to induce VSMC aging. ChIP-seq experiments revealed PLA expression induced a rapid redistribution of H3K9me3 and H3K27me3 occupancy in the genome, rather than an indiscriminate loss of repressive domains. By cross-analyzing differentially expressed genes with peak annotations from ChIP-seq data, we identified key targets and pathways that might be susceptible to changes in the epigenetic landscape induced by loss of heterochromatin. Pathway analysis of the upregulated gene set revealed that insulin signaling ranked top of involved biological processes followed by regulation of protein and lipid clearance via autophagy-related processes. Importantly, deregulation of these same pathways was reiterated in vivo in the mouse model of inducible mPLA expression suggesting that loss of heterochromatin can instigate early loss of homeostatic mechanisms potentially impacting downstream pathology. Indeed, the pathways identified have all been implicated in aging and calcification.^49^ For example, medial arterial calcification is a common complication in type 2 diabetes and insulin signaling has been implicated directly in the maintenance of VSMC phenotype and protection from vascular calcification.^50–53^ Likewise, autophagy and lipid metabolism impact vascular calcification via multiple mechanisms.^49,54^

Notably, the subset of downregulated genes with associated increases in repressive histone marks were enriched in DNA-binding proteins involved in transcriptional regulation and the DNA damage response (DDR). These included NFKB1, the absence of which has been shown to exacerbate DNA damage in mouse models of stress.^55^ It is plausible that epigenetic silencing of DNA damage repair genes results in the genome remaining unrepaired leading to activation of upstream DNA damage sensing ATM (ataxia-telangiectasia mutated) kinase, which mediates crosstalk between the DDR and regulation of the SASP.^13,56,57^ Our data further suggests that loss of heterochromatin epigenetically modulated key factors downstream of the ATM kinase, including the stress response kinase HIPK2, which showed elevated expression. HIPK2 can be activated or repressed by DNA damage and can modulate several critical inflammatory pathways including NF-κB.^58,59^ It has also been identified in unbiased approaches as an upstream regulator of TGFβ/Smad3 (mothers against decapentaplegic homolog 3), Wnt, p53, and NF-κB during kidney fibrosis with all these pathways also implicated in VSMC osteogenic differentiation.^34^ Importantly, vascular calcification is tightly associated with accrual of DNA damage^15,16,60,61^ and blockade of ATM signaling in VSMCs overexpressing PLA or exposed to elevated calcium and phosphate can attenuate osteogenic differentiation and abrogate production of pro-osteogenic SASP cytokines.^13,15,16^ Importantly, the DDR also drives epigenetic modifications, and further studies are now needed to determine the precise timeline of activation of DDR signaling and the role of novel regulators such as HIPK2 in vascular inflammation and calcification.

The mouse model of inducible PLA expression also replicated the pathways seen in vitro including DNA damage and chemokine signaling potentially indicative of early SASP activation. In this model, we also observed increased expression of p16, Runx2, pro-osteogenic cytokines including BMP2, IL6, and IL8 and other calcification regulators coinciding with heterochromatin loss but preceding detection of overt DNA damage indicated by γH2AX staining. This is consistent with heterochromatin loss leading to early derepression of key osteogenic and inflammatory pathways and further work is now required to examine details of the epigenetic regulation of these genes.

### Epigenetic Change Links to Inflammaging

A key finding in this study was that inflammation was a very early response to mineral stress preceding mineralization. VSMCs secreted chemokines and growth factors known to be elevated in the serum of calcified patients and previously implicated in driving calcification including HGF and IL1β, which were activated early and persisted throughout calcification.^62–64^ Multiple chemokines were also activated in concert potentially because of their physical clustering in the genome and further investigation of the role of chemokines in vascular calcification is clearly warranted. VSMCs treated with inhibitors of the methyltransferases SUV39H1 and EZH2 showed an enhanced cytokine profile and accelerated calcification supporting a role for heterochromatin loss in driving inflammatory pathways consistent with a previous study that proposed the H3K9me3 mark and SUV39H1 can maintain a sustained proinflammatory phenotype in VSMCs from diabetic mice.^65^

Interestingly, in CKD, we observed that H3K27me3 was the earliest affected histone mark in VSMCs. Moreover, its loss in response to mineral dysregulation both in vitro and in CKD rats preceded the accumulation of PLA. H3K27me3 is regulated by EZH2 which has been shown to play a crucial role in protecting VSMCs against autophagic cell death and in the regulation of expression of stiffness associated genes. EZH2 also acts as a suppressor of osteoblast differentiation in bone with all these pathways also associated with calcification and deregulated in response to PLA.^66–69^ The factors that regulate the expression of epigenetic modifiers are combinatorial and largely unknown but increased MR (mineralcorticoid receptor) signaling was shown to reduce EZH2 in aging mice.^67^ Importantly MR signaling has been strongly implicated in regulating vascular calcification in response to phosphate in vitro and in vivo in animal models and patients with CKD.^70,71^ More in-depth analyses of how different epigenetic modifiers are regulated by environmental factors might eventually allow the actualization of epigenetic rejuvenation or reversal of detrimental epigenetic memory to reduce or delay pathology.^72^

The elimination of senescent cells has also been shown to have beneficial effects on the life span and health span of mice.^4,73,74^ Our data suggests that capturing or modifying primed cells during presenescence may also be beneficial and that PLA–expressing cells may represent this primed population. We observed PLA accumulation in response to mineral stress at the earliest stages of calcification both in vitro and in vivo where it is likely to rapidly accelerate multiple aging pathways in a feed-forward cycle. Further understanding of the pathways that promote nuclear lamina dysfunction in VSMCs is also clearly warranted.

### Limitations

There are some limitations of this study related to the human tissue samples evaluated. Vessels from children with CKD were from different vascular beds from controls; therefore, this may have an impact on heterochromatin levels. Much of the human histological data although significant is based on relatively small sample sizes, and it will be important to validate specific findings in larger patient cohorts. A major limitation of the rat CKD model is that calcification, once initiated, is rapid and severe, and many cells die in the process so it is difficult to draw direct comparisons with human CKD where exposure is long-term and calcification incremental. In the rat model, it would be possible to miss longer-term changes, which may account for why we were unable to quantify the loss of H3K9me3 in calcified areas in the rat model.

## ARTICLE INFORMATION

### Acknowledgments

Microscopy was performed at the Wohl Cellular Imaging center, King’s College London. Sequencing was performed at the Genomics Research Platform King’s College London. Graphical abstract and schematic were created with BioRender.com.

### Author Contributions

C.Y. Ho and C.M. Shanahan contributed to the conception; C.Y. Ho, M.-Y. Wu, and C.M. Shanahan to experimental design; C.Y. Ho, M.-Y. Wu, J. Thammaphet, S. Ahmad, L. Draganova, G. Anderson, A. Verhulst, and R. Hayward to the acquisition of data; C.Y. Ho, M.-Y. Wu, J. Thammaphet, J. Ho C.S, U.S. Jonnalagadda, R. Shroff, W.T.L. Wen, R. Foo, and C.M. Shanahan to analysis and interpretation of data. C.Y. Ho, M.-Y. Wu, and C.M. Shanahan wrote and revised the manuscript and all authors provided final approval of the submitted version.

### Sources of Funding

This work was supported by British Heart Foundation Programme Grants (RG/17/2/32808 and RG/F/21/110064) to C.M. Shanahan.

### Disclosures

None.

### Supplemental Material

Supplemental Methods

Tables S1–S4

Figures S1–S9

Data Set

Major Resources Table

References 37,38,75–88
